# Comparative Analysis of Thrips Prey and Pollen Usage by Three Predatory Mites, *Amblyseius largoensis*, *Neoseiulus californicus* and *Neoseiulus barkeri*

**DOI:** 10.3390/insects17101074

**Published:** 2026-10-17

**Authors:** Asim Munawar, Li Kaiyu, Chen Xuan, Amr S. Abou El-Ela, Zengrong Zhu, Xiaoduan Fang, Zhizhi Wang, Jianwei Fu, Ruibin Xu, Wenwu Zhou

**Affiliations:** 1 State Key Laboratory of Rice Biology and Breeding, Ministry of Agriculture and Rural Affairs Key Laboratory of Molecular Biology of Crop Pathogens and Insect Pests, Institute of Insect Sciences, Zhejiang University, Hangzhou 310058, China; asim_munawar@zju.edu.cn (A.M.); 22416091@zju.edu.cn (L.K.); 12116104@zju.edu.cn (C.X.); zrzhu@zju.edu.cn (Z.Z.); zzwang0730@zju.edu.cn (Z.W.); 2 Plant Protection Department, Faculty of Agriculture (Saba Basha), Alexandria University, Alexandria 21531, Egypt; 3 Guangdong Key Laboratory of Animal Conservation and Resource Utilization, Guangdong Public Laboratory of Wild Animal Conservation and Utilization, Institute of Zoology, Guangdong Academy of Sciences, Guangzhou 510260, China; fangxiaoduan@163.com; 4 Institute of Crop Sciences, Fujian Academy of Agricultural Sciences, Fuzhou 350003, China; fjw9238@163.com; 5 Institute of Quality Standards and Testing Technology for Agro-Products, Fujian Academy of Agricultural Sciences, Fuzhou 350013, China; 6 College of Food Science, Fujian Agriculture and Forestry University, Fuzhou 350002, China

**Keywords:** Phytoseiidae, augmentative biological control, intraguild competition, prey-stage selectivity, alternative food, *Thrips tabaci*

## Abstract

Phytoseiid mites (PDMs) utilize both herbivorous prey and an alternative food source in agroecosystems. However, biocontrol screening of PDMs has largely focused on their predation function against prey, with their use of alternative food often ignored. Therefore, in the present study, we compared the performance of three PDM species (*Amblyseius largoensis* (Muma), *Neoseiulus californicus* (McGregor) and *Neoseiulus barkeri* Hughes), fed on two food sources: onion thrips (*Thrips tabaci*) and pollen (an alternative food) on potato plants. All three PDMs concentrated their attacks on the vulnerable prey stages, with early larval instars attacked at the highest rate, while *N. californicus* proved the most effective egg predator and *N. barkeri* the least. *A. largoensis* located and attacked prey the fastest both in leaf arenas and on intact plants, despite the longest per-prey handling time and attacked the fewest prey across a density gradient while leaving the least thrips-induced leaf damage. Prey rather than pollen was the primary driver of survival in every PDM, and *A. largoensis* persisted longest under food limitation and was the only species to gain further benefit from pollen when prey was already available. This ranking inverted in mixed-species arenas, where *N. californicus* consistently outsurvived the other two species under prey-present and pollen-only conditions. Foraging efficiency, food resource use and intraguild dominance were dissociated for PDM performance. Thus, a multiple-trait screening can be considered in PDM selection for thrips management in crops.

## Simple Summary

Onion thrips are minute insects that feed on potato leaves, scarring the foliage, reducing yields and spreading plant viruses. Growers have long relied on chemical sprays to control them, but thrips have become resistant to many of these chemicals, and the sprays also harm the beneficial insects that would otherwise help keep pests in check. One alternative is to release tiny predatory mites that hunt thrips on the crop. Growers can only buy a handful of predatory mite species, and these are usually chosen based on how many pests they eat, even though these predators also feed on pollen when prey is scarce and are often released together with other predator species. We compared three predatory mite species on potato plants, measuring how quickly each found and attacked thrips, how long each survived on thrips, on pollen and on both together, and how each fared when sharing a leaf with another predator species. No single species was best at everything. The fastest hunter survived longest when food was scarce and was the only one to benefit from having pollen alongside prey, yet it lost out when a competing predator was present. Choosing the right predator therefore means testing several different abilities, not just counting how many pests it eats.

## 1. Introduction

Insect pests remain one of the most persistent threats to global food security, with pest-related losses continuing to constrain crop productivity across major agricultural systems despite decades of management effort [1,2,3]. Potato (*Solanum tuberosum* L.), one of the most widely cultivated food crops worldwide and a critical source of caloric and nutritional security in both developed and developing regions, is particularly vulnerable to a broad complex of insect pests that reduce yield and tuber quality [4,5]. Among these, the onion thrips, *Thrips tabaci* Lindeman (Thysanoptera: Thripidae), has emerged as a particularly damaging generalist herbivore of potato, causing direct feeding injury, leaf silvering, chlorotic and necrotic lesions and stunted growth, while also serving as a vector of economically important plant viruses [6,7]. The polyphagous habit of *T. tabaci*, combined with its cryptic feeding behavior, short generation time and capacity for rapid population growth under favorable conditions, makes it a notably difficult pest to manage using conventional approaches, and outbreaks continue to impose substantial economic losses on potato growers worldwide [8].

Management of *T. tabaci* and other thrips species has historically relied heavily on repeated applications of broad-spectrum synthetic insecticides [9]. This reliance has produced a range of well-documented negative consequences. Thrips populations, including *T. tabaci*, have repeatedly evolved resistance to multiple insecticide classes within relatively few growing seasons, substantially eroding the long-term efficacy of chemical control programs [10,11,12]. Beyond resistance, intensive insecticide use poses considerable risks to human health, non-target organisms and broader ecosystem function and frequently disrupts populations of naturally occurring predators and parasitoids that would otherwise contribute to pest suppression, reinforcing a cycle of dependence on chemical inputs [13,14]. These concerns are compounded by shifting consumer demand for produce with minimal pesticide residues and by increasingly stringent regulatory restrictions on insecticide registration in many potato-producing regions [15]. Collectively, these pressures have created an urgent need for effective, environmentally sustainable alternatives capable of suppressing thrips populations while reducing reliance on synthetic inputs.

Biological control using natural enemies represents one of the most promising alternatives to conventional insecticide-based thrips management [16,17]. Among the natural enemies available for thrips control, predatory mites (PDMs) of the family Phytoseiidae have received considerable attention owing to their small size, high reproductive potential, capacity to feed on multiple thrips developmental stages and, in many PDMs, the ability to subsist on alternative food sources such as pollen when prey availability is low [18,19]. Several phytoseiid PDMs are now produced and marketed commercially for augmentative release against thrips and other arthropod pests in protected and open-field cropping systems [20]. Phytoseiids differ in the breadth of their diets, with generalist species able to persist for long periods on pollen and other non-prey resources [18,21]. The extent to which pollen substitutes for prey differs among phytoseiids, however, and whether a species can exploit both resources together, rather than merely tolerating pollen in the absence of prey, has direct consequences for how long a released population persists before pest pressure builds. Screening of candidate agents has nonetheless concentrated on predation rate against the target pest, with alternative food use assessed separately if at all.

However, the set of PDMs available commercially is small relative to the diversity of the family, and its composition reflects mass-rearing feasibility, historical precedent and cost as much as demonstrated performance in any particular crop–pest system [22,23]. Selection of an agent for a given system is therefore often guided by what can be purchased rather than by system-specific evaluation of predatory capacity, environmental resilience or compatibility with the target pest and cropping conditions. Indigenous phytoseiid faunas, by contrast, include numerous PDMs that occur naturally in agricultural landscapes yet have never been assessed as candidate agents, and the few studies that have evaluated such PDMs report performance comparable to established commercial agents [24,25]. A further consideration, rarely addressed at the screening stage, is how candidate PDMs interact with one another. Augmentative programs increasingly deploy more than one PDM, yet phytoseiids compete directly where their diets overlap, and the outcome of such interactions need not follow from the performance of each PDM measured in isolation [26,27]. Whether the traits that determine predatory efficiency also determine competitive standing within a mixed guild is therefore a practically consequential question that single-PDM screening cannot answer.

Here we compare three phytoseiid PDMs—*Amblyseius largoensis* (Muma), * Neoseiulus californicus* (McGregor) and *Neoseiulus barkeri* Hughes—as candidate agents against *T. tabaci* on potato plants. *N. californicus* and *N. barkeri* are established commercial agents deployed across a range of cropping systems, whereas *A. largoensis*, although recorded from the phytoseiid fauna of southern China, has not been developed for augmentative release against thrips [28,29]. *A. largoensis* was selected because it occurs naturally in the region, is widely distributed across tropical and subtropical zones and has not been evaluated against thrips on potato. We asked whether predatory performance measured in isolation predicts how these PDMs perform when food resources vary and when they occur together. To address this, we evaluated stage-specific prey attack and foraging behavior under confined and whole-plant conditions, survival under prey, pollen and their combination, and interactions among the three PDMs when sharing an arena under prey-present and prey-absent conditions. Together these assays test whether the traits that determine predatory efficiency also determine performance under resource limitation and within a mixed guild and therefore whether single-assay screening is a sound basis for agent selection.

## 2. Materials and Methods

### 2.1. Biological Materials and Experimental Conditions

Onion thrips, *T. tabaci*, served as the target prey in all predation and behavioral assays. The founding population was collected in 2025 from field-grown cabbage at the Zijingang Campus of Zhejiang University, Hangzhou, China and established as a laboratory colony at the Institute of Insect Sciences, Zhejiang University. The colony was reared on potato plants for multiple generations before experimental use to minimize effects of field acclimation and to obtain a stable laboratory population [8]. Three phytoseiid PDMs were used: *A. largoensis*, *N. californicus* and *N. barkeri*. *N. californicus* was obtained from a commercial producer (Fujian Yanxuan Biological Control Technology Co., Ltd., Fujian, China; distributed by Hangzhou Guoyou Agricultural Production Materials Co., Ltd., Hangzhou, China). *A. largoensis* was originally collected from *Ficus benjamina* at Qiongtai Normal University, Hainan, and *N. barkeri* was originally obtained as a commodity population from Chaozhou Zhongtian Agricultural Co., Ltd.; both have since been maintained in continuous culture for more than ten generations at the Institute of Zoology, Guangdong Academy of Sciences, Guangzhou, China. Colony provenance and PDM identity are as described previously [19]. *A. largoensis* was reared on the mite *Carpoglyphus lactis* (Linnaeus) and *N. barkeri* on the mite *Aleuroglyphus ovatus* (Troupeau), each on a wheat bran carrier. Adult female mites were used in all assays. Unless otherwise stated, all experiments were conducted at 25 ± 1 °C, 70 ± 5% relative humidity and a 16 h:8 h (light: dark) photoperiod under fluorescent lighting.

Potato plants, *Solanum tuberosum* L. (*cv. Lishu*), were grown at Zhejiang University, Hangzhou, Zhejiang Province, China. Plantlets were propagated in vitro by tissue culture and maintained at 20 ± 1 °C under a 16 h photoperiod at a light intensity of 100 μmol m^−2^ s^−1^ using LED T5 lamps. Four weeks after sub-culturing, plantlets were transferred to pots containing peat-based potting soil and maintained in a glasshouse under a 16 h:8 h (light: dark) cycle at 25 ± 2 °C (light) and 20 ± 2 °C (dark), with a light intensity of 600–800 μmol m^−2^ s^−1^. Plants were watered daily, and three-week-old plants were used for thrips maintenance and all experimental assays [30].

### 2.2. Stage-Specific Prey Attack and Predatory Behavior

Stage-specific prey attack and predatory behavior were assessed in detached potato leaf arenas prepared in Petri dishes (90 mm × 15 mm), each containing a single intact potato leaf with the petiole wrapped in moist cotton to maintain turgidity and reduce desiccation. Newly emerged *T. tabaci* female adults of uniform size and condition, drawn from the same cohort of eggs, were used throughout so that age and physiological state were standardized across PDMs. Unless otherwise stated, females were starved for 24 h before release and each PDM species was tested in isolation under the environmental conditions described in Section 2.1, with five replicates per species [18].

#### 2.2.1. Stage-Specific Prey Attack

Five exposed developmental stages of *T. tabaci* were offered simultaneously: first-instar larvae, second-instar larvae, prepupae, pupae and adults. Five individuals of each stage were released onto a single leaf arena, giving 25 prey per arena, and allowed to settle for 12 h before five PDMs of a single species were introduced. After 24 h the number of surviving thrips of each stage was recorded and the number attacked was calculated as the difference between the initial and final numbers of individuals of that stage. Because prepupae and pupae may molt or leave the leaf surface during the assay, values for these two stages represent apparent rather than absolute attack.

#### 2.2.2. Egg Consumption

Egg predation was assessed separately because *T. tabaci* oviposits into potato leaf tissue [31]. Eight male–female pairs of adult *T. tabaci* were released onto an intact potato leaf and allowed to oviposit for 48 h, after which adults were removed and the initial number of eggs was counted under a stereo microscope (ZEISS/OPLENIC digital imaging system, 1.0×; working distance 81 mm, Carl Zeiss Microscopy GmbH, Jena, Germany). Three 24 h-starved PDMs of a single species were then released onto each egg-bearing arena and allowed to feed for 24 h. Predators were removed and leaves were held at 20 ± 1 °C to permit hatching, after which the numbers of hatched larvae and unhatched eggs were recorded. Egg consumption was calculated as the initial egg number minus the sum of larvae hatched and eggs remaining unhatched. No predator-free control was included, so this estimate assumes that egg loss from causes other than predation was negligible. Because *T. tabaci* inserts its eggs into leaf tissue, predation was limited to the exposed portion of the chorion at the oviposition site; eggs were not manipulated or exposed artificially.

#### 2.2.3. Prey Density Response

The effect of prey availability on consumption was assessed using first-instar *T. tabaci* larvae, the stage attacked at the highest rate by all three PDMs in the stage-preference assay, at five initial densities: 5, 10, 20, 30 and 40 larvae per arena. Larvae at the assigned density were released onto a detached potato leaf arena and allowed to settle for 24 h. Prior to the assay, adult female PDMs were starved for 24 h to standardize their feeding motivation. Two starved adult females of a single predator species were then introduced into each arena. After 48 h, surviving larvae were counted, and the number of prey attacked was calculated as the difference between the initial and final numbers of larvae. Leaf area damaged by thrips feeding was quantified from digital images of each leaf using ImageJ (v.1.54f), as a measure of the crop protection achieved by each PDM [32]. Ten replicates were performed for each predator × prey density combination.

#### 2.2.4. Foraging Behavior

Behavioral parameters of each PDM were recorded using first-instar *T. tabaci* larvae as prey, with each mite observed continuously from release under a stereomicroscope (ZEISS/OPLENIC digital imaging system, 1.0×; working distance 81 mm). For handling time, a single larva was placed on a leaf arena followed by one PDM; handling time was recorded as the interval between prey capture and release of the prey by the predator [33]. Searching time and the two latency measures were recorded in parallel arenas: searching time was the interval between predator release and location of the prey, time to first contact the interval until first physical contact with a larva and time to first attack the interval until the first clear attack attempt, including grasping, piercing or feeding. Five replicates were performed per PDM.

### 2.3. Predator Survival Assays Under Varying Food Availability

Survival of *A. largoensis*, *N. californicus* and *N. barkeri* was assessed under four food-resource treatments: no pollen and no thrips; pollen only; thrips only; and pollen plus thrips. For each replicate, 10 PDMs of a single species were released onto a detached potato leaf arena prepared as described in Section 2.2. Potato flower anthers served as the pollen source, based on prior confirmation that the PDMs tested can use potato flower pollen as an alternative food [18]. In prey-containing treatments, 50 first-instar *T. tabaci* larvae were provided. Pollen and prey were renewed every 2 days according to treatment, and dead mites and prey residues were removed at each observation. Surviving mites were counted every 2 days for 30 days, and survival was expressed as the percentage of the initial 10 mites remaining. Each PDM was tested separately, with five replicate arenas per PDM × treatment combination (3 PDMs × 4 treatments × 5 replicates = 60 experimental units).

### 2.4. Interspecific Competition Under Shared-Resource Conditions

Interspecific competition assays were conducted to evaluate the performance of adult PDM species when sharing the same leaf arena and food resources. The assays were designed to assess competitive interactions among adults rather than stage-specific intraguild predation. The same developmental stage was used across species to standardize predator age and functional status, thereby preventing developmental-stage differences from confounding species-level competitive effects. Competitive performance was assessed from species-specific survival under shared-resource conditions and from the number of prey attacked when thrips were available. Four combinations were established: T1, *A. largoensis* + *N. californicus*; T2, *A. largoensis* + *N. barkeri*; T3, *N. barkeri* + *N. californicus*; and T4, all three PDMs together. For each replicate, 30 first-instar *T. tabaci* larvae were released onto a detached potato leaf arena and allowed to settle for 24 h before predator release. Five PDMs of each species were introduced into the same arena, giving a total of 10 mites in the two-PDM combinations and 15 in the three-PDM combination. After 48 h, surviving thrips were counted and the number attacked was calculated from the initial number released; surviving PDMs of each species were counted separately. A parallel assay used the same four combinations with no thrips present and potato flower anthers supplied as the sole food resource. After 48 h, surviving PDMs of each species were counted separately. At the end of each assay, surviving PDMs were collected and preserved in 75% ethanol, after which microscope slides were prepared, and species identity was confirmed based on diagnostic morphological characteristics under a microscope. Ten replicates were performed for each combination under each condition.

### 2.5. Predator Foraging Behavior on Potato Plants

Foraging behavior was assessed on three-week-old potted potato plants under glasshouse conditions [18]. To minimize interference from soil or substrate-derived cues, the pot surface was covered with aluminum foil, and the basal stem region was wrapped with moist cotton to prevent predator escape during observation. On each plant, one mid-canopy leaf was selected as the prey site and infested with five first-instar *T. tabaci* larvae 24 h before predator release. For each trial, one starved PDM was released at the base of the stem and followed continuously by direct visual observation, with a hand lens used where closer inspection was required to confirm contact with prey and the onset of an attack. Time to first prey encounter was recorded as the interval from release to first physical contact with a thrips larva and time to first attack as the interval from release to the first clear attack attempt, including grasping, piercing or feeding. Observation ended once the first attack was recorded and the mite was removed; prey was therefore not consumed during the assay. Mites that failed to locate prey within 30 min were classified as non-responders and excluded from the time-based analyses; additional individuals were tested until eight responding individuals were obtained for each PDM species. The assay was arranged in eight replicate blocks, with one individual of each PDM species tested independently on a separate plant per block, resulting in eight responding individuals per species and 24 plants in total. Four blocks were conducted on each of two consecutive days, with all three PDM species represented equally on both days.

### 2.6. Statistical Analysis

Analyses were performed in R (version 4.1.0; R Core Team) and SPSS (version 16.0; SPSS Inc., Chicago, IL, USA). Figures were produced using ggplot2 within the tidyverse, and post hoc comparisons were computed using emmeans with Tukey adjustment. Significance was assessed at *p* ≤ 0.05 throughout. Continuous response variables—prey handling time, searching time, time to first contact and time to first attack in leaf arenas and time to first prey encounter and time to first attack on whole plants—were analyzed by one-way analysis of variance with PDMs as a fixed factor, followed by Tukey’s HSD test where significant effects were detected. Residual normality was assessed by the Shapiro–Wilk test and homogeneity of variance by Levene’s test, using the car package. Count and proportional data were analyzed by generalized linear models (GLMs) fitted according to the structure of the response variable: binomial models for responses measured as events out of a fixed total and Poisson models for counts. Over-dispersion was assessed from the Pearson residual dispersion ratio and quasi-binomial or quasi-Poisson families were fitted where over-dispersion was detected. Likelihood-ratio χ^2^ tests are reported for binomial and Poisson models and F tests for the corresponding quasi-family models fitted where over-dispersion was detected. This approach was applied to stage-specific prey attack, initial and consumed egg numbers and total prey attacked and predator survival in the interspecific interaction assays. Prey attacked and leaf area damaged in the prey-density assay were analyzed separately at each prey density by one-way analysis of variance with PDMs as a fixed factor, followed by Tukey’s HSD test. Predator survival under the four food treatments was analyzed using a linear mixed-effects model with PDMs, food treatment, time and their interactions as fixed effects and replicate as a random effect. Significance was assessed by Type III ANOVA with Satterthwaite’s approximation, followed by estimated marginal means with Tukey-adjusted pairwise comparisons.

## 3. Results

### 3.1. Prey-Stage Selectivity, Egg Predation and Density-Dependent Predation by Three Phytoseiid Predators

**Figure 1:**
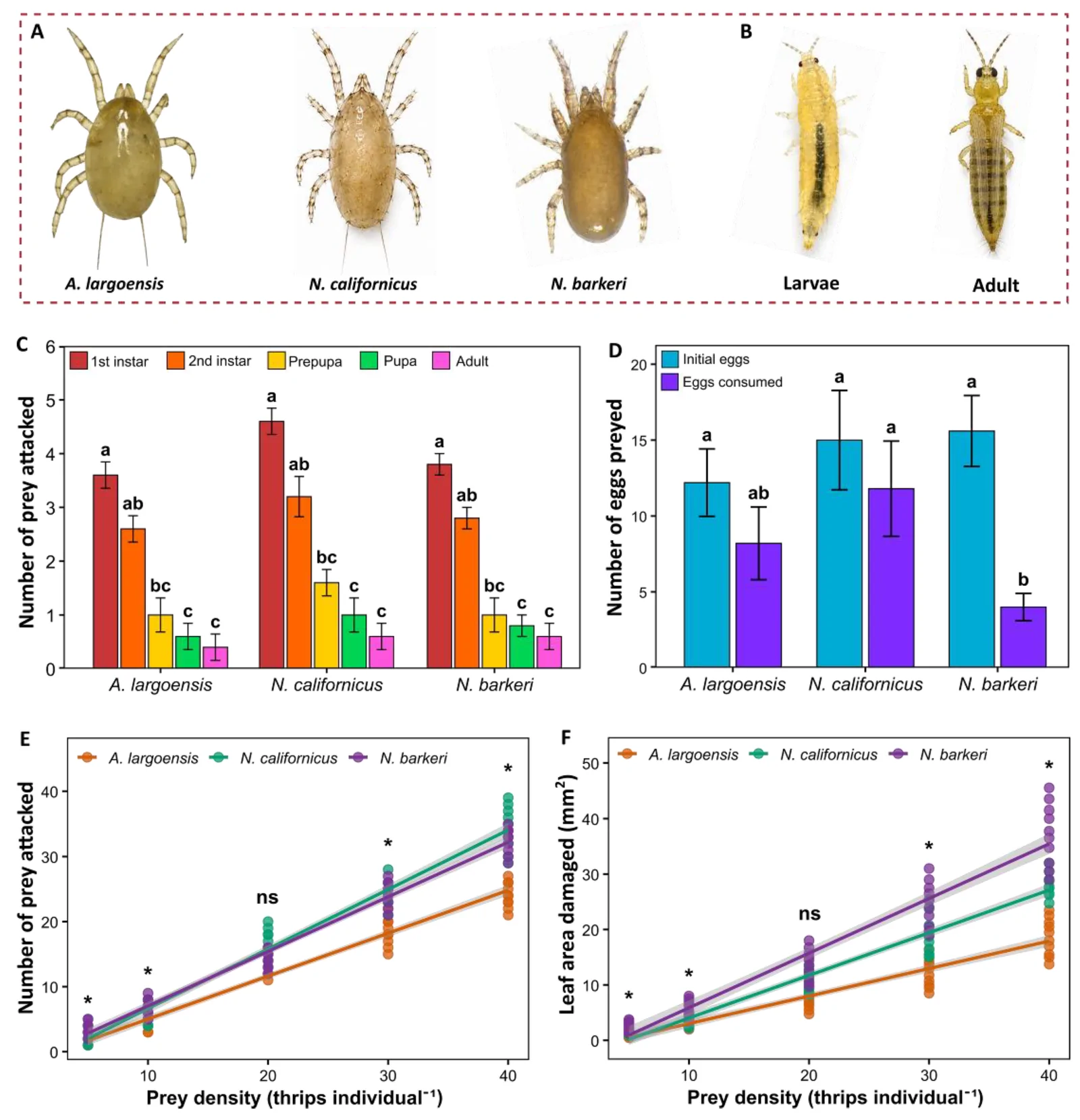
Prey-stage selectivity, egg predation and density-dependent predation by three phytoseiid PDMs on *T. tabaci*. (**A**) Dorsal micrographs of the three PDMs used throughout the study: *A. largoensis*, *N. californicus* and *N. barkeri*. (**B**) Reference images of *T. tabaci* larvae and adults. (**C**) Number of prey attacked by each PDM when five developmental stages of *T. tabaci* were offered simultaneously (*n* = 5 replicates per PDM). (**D**) Initial number of eggs offered and number of eggs consumed by each PDM (*n* = 5 replicates per PDM). (**E**) Number of prey attacked (**F**) leaf area damaged by each PDM across five prey densities (*n* = 10 replicates per species at each density). Bars and points show mean ± SE. In (**C**,**D**), different letters indicate significant differences among the bars shown, analyzed by GLM (binomial or quasi-binomial as appropriate) followed by Tukey-adjusted pairwise comparisons. In **(E**,**F**), asterisks indicate a significant difference among the three PDMs at that density; pairwise comparisons are reported in the text analyzed by ANOVA followed by Tukey’s HSD performed separately at each prey density (* *p* < 0.05; ns = not significant).

The three PDMs were readily distinguished by body shape and coloration (Figure 1A) and were tested against *T. tabaci* larvae (Figure 1B). The number of prey attacked declined progressively with increasing developmental stage across all three PDMs (GLM: *A. largoensis*, χ^2^_4_ = 36.62, *p* < 0.001; *N. californicus*, χ^2^_4_ = 50.16, *p* < 0.001; *N. barkeri*, χ^2^_4_ = 36.15, *p* < 0.001; Figure 1C). First-instar larvae were attacked far more frequently than second-instar larvae; prepupae, pupae and adults were attacked considerably less often than either larval stage. This shared preference for early instars likely reflects the smaller body size, thinner cuticle and reduced defensive capacity of younger prey.

Egg predation revealed a clear species difference: the number of eggs initially available did not differ among species, confirming equivalent starting conditions across arenas (*F*_2_,_12_ = 0.48, *p* = 0.628). *N. californicus* consumed nearly all the eggs it was offered, with no significant difference between the initial and consumed counts, identifying it as the most effective egg predator of the three PDMs. *N. barkeri* showed the opposite pattern: it consumed significantly fewer eggs than the number offered and significantly fewer than *N. californicus* consumed (*p* = 0.037), indicating that most eggs presented to this species survived predation. *A. largoensis* was intermediate, consuming a number of eggs that did not differ significantly from either the initial count or from *N. californicus* consumption (Figure 1D). *N. californicus* was therefore the strongest egg predator of the three PDMs; *N. barkeri* was comparatively ineffective against this life stage, despite performing similarly to *A. largoensis* against older larval instars. Prey attacked and leaf area damaged both increased with prey density across all three PDMs (Figure S1). Species differed significantly from one another at every density level tested, though the identity of the most effective predator shifted with density. At the lowest density, *N. barkeri* attacked significantly more prey than either *A. largoensis* or *N. californicus*, which did not differ from each other. At 10-prey, both *N. californicus* and *N. barkeri* attacked significantly more prey than *A. largoensis*, again without differing from one another. By 20-prey, *N. californicus* became the top performer, significantly exceeding both other species, while *A. largoensis* and *N. barkeri* converged to statistically indistinguishable attack rates. At the two highest densities, *N. californicus* and *N. barkeri* attacked comparable numbers of prey, both consistently and substantially exceeding *A. largoensis* (density: 5-prey, *F*_2,27_ = 15.73; 10-prey, *F*_2,27_ = 10.66; 20-prey, *F*_2,27_ = 12.61; 30-prey, *F*_2,27_ = 26.93; 40-prey, *F*_2,27_ = 47.78; *p* < 0.001; Figure 1E). Across the density gradient, *A. largoensis* consistently ranked at or near the bottom for raw prey attacked, never exceeding either of the other two species at any density tested.

Leaf area damaged followed a related but not identical trajectory (Figure 1F). At the two lowest densities, thrips-induced leaf damage closely mirrored the attack pattern: damage did not differ significantly between arenas containing *A. largoensis* and those containing *N. californicus*, while arenas containing *N. barkeri* sustained significantly greater damage than both. Leaf damage was consistently lowest in arenas containing *A. largoensis*, intermediate with *N. californicus* and highest with *N. barkeri*, across every density from 20-prey upward (density: 5-prey, *F*_2,27_ = 10.94; 10-prey, *F*_2,27_ = 20.61; 20-prey, *F*_2,27_ = 21.38; 30-prey, *F*_2,27_ = 31.07; 40-prey, *F*_2,27_ = 54.63; *p* < 0.001). This pattern did not simply track prey attacked, however: *A. largoensis* and *N. californicus* attacked statistically similar numbers of prey at the two highest densities, yet arenas containing *N. californicus* sustained significantly greater damage than those containing *A. largoensis* at both, a discrepancy suggesting that feeding behavior per prey item, not attack frequency alone, determines how much feeding time thrips retain before being intercepted and consequently how much leaf damage they inflict.

### 3.2. Foraging Behavior of Three Phytoseiid Predators Under Confined and Whole-Plant Conditions

**Figure 2:**
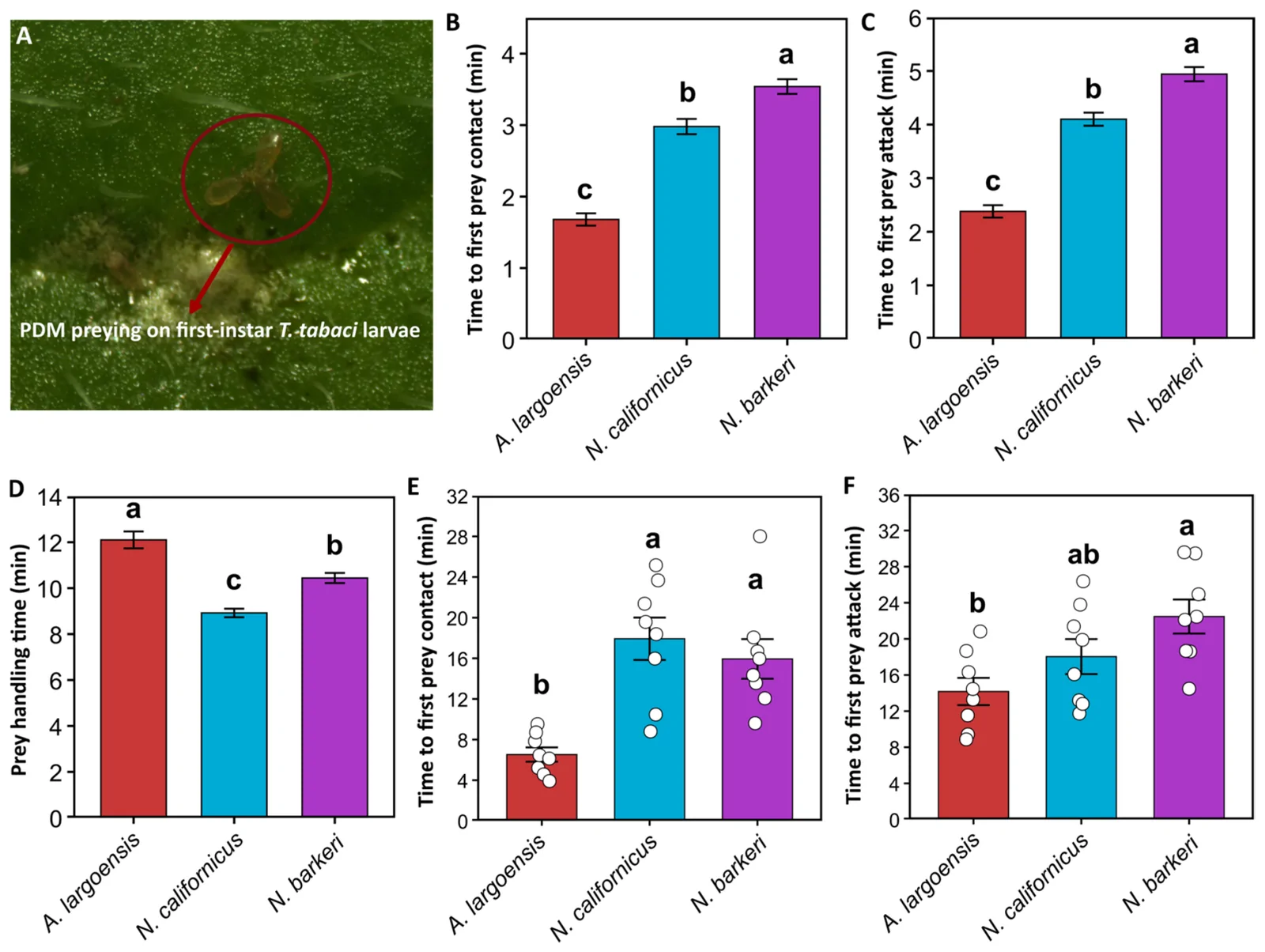
Foraging behavior of three PDMs on *T. tabaci* under confined and whole-plant conditions. (**A**) PDMs preying on a first-instar *T. tabaci* larva during confined-arena assays. (**B**) Latency to first physical contact with prey following release. (**C**) Latency to first attack following release. (**D**) Prey handling time per capture event (*n* = 5 replicates per PDM). (**E**) Time to first prey contact and (**F**) time to first attack under whole-plant, glasshouse conditions, following release onto thrips-infested potato plants (*n* = 8 responding individuals per PDM). Bars show mean ± SE; white points in (**E**,**F**) show individual replicates. Different letters above bars indicate significant differences among the three PDMs within each panel. Data in (**B**–**D**) were analyzed by GLM followed by Tukey-adjusted pairwise comparisons; data in (**E**,**F**) were analyzed by ANOVA followed by Tukey’s HSD (*p* < 0.05).

We compared the foraging behavior of *A. largoensis*, *N. californicus* and *N. barkeri* under confined Petri-dish conditions, using a thrips-infested leaflet as the arena (Figure 2A). PDMs differed significantly in latency to first physical contact with prey (*F*_2,12_ = 92.91, *p* < 0.001), latency to first attack (*F*_2,12_ = 111.06, *p* < 0.001), prey handling time (*F*_2,12_ = 34.81, *p* < 0.001) and pre-encounter searching time (*F*_2,12_ = 69.41, *p* < 0.001; Figure S2). *A. largoensis* was consistently the fastest of the three species across all latency measures and *N. barkeri* the slowest, with *N. californicus* intermediate (Figure 2B,C). Handling time followed a different ranking, with *A. largoensis* retaining each prey item longest, *N. californicus* releasing prey soonest and *N. barkeri* again intermediate (Figure 2D). *A. largoensis’s* advantage therefore lay specifically in prey location and attack initiation, not in the speed of subduing and consuming prey once contact had been made.

We then tested whether this ranking held under whole-plant conditions, where predators located infested foliage across intact plant architecture rather than a confined arena. PDMs again differed significantly in time to first prey contact (*F*_2,21_ = 12.87, *p* < 0.001). *A. largoensis* located prey substantially faster than either *N. californicus* or *N. barkeri*, which did not differ from one another (Figure 2E). Time to first attack followed the same pattern (*F*_2,21_ = 5.41, *p* = 0.013): *A. largoensis* fastest, *N. californicus* intermediate, *N. barkeri* slowest (Figure 2F). The ranking observed in confined arenas was therefore preserved on intact plants, indicating that *A. largoensis’s* foraging-speed advantage is not an artifact of simplified laboratory conditions.

### 3.3. Survival of PDMs Under Varying Prey and Alternative Food Availability

**Figure 3:**
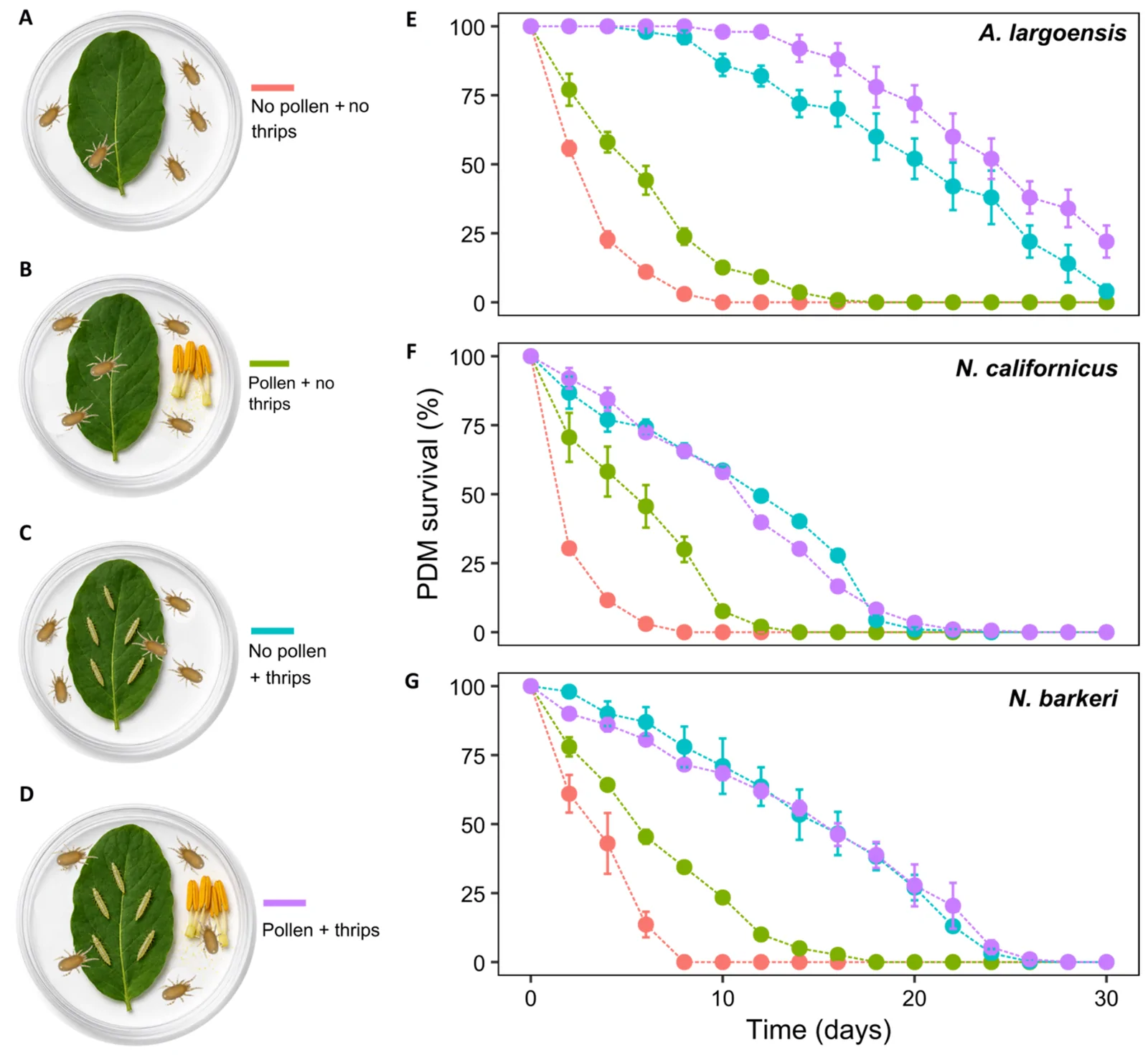
Survival of PDMs under combinations of prey and alternative food availability. (**A**–**D**) Experimental arenas illustrating the four food treatments: (**A**) no pollen and no thrips; (**B**) pollen supplied in the absence of thrips prey; (**C**) thrips prey supplied in the absence of pollen; and (**D**) combined availability of pollen and thrips prey. (**E**–**G**) Survival curves under each treatment, presented separately for (**E**) *A. largoensis*; (**F**) *N. californicus*; and (**G**) *N. barkeri*. Ten PDMs were released per arena and survivors counted every 2 days for 30 days; survival is expressed as the percentage of the initial 10 PDMs remaining. *n* = 5 replicate arenas per PDM × treatment combination. Points and error bars represent mean ± SE. Data were analyzed using a linear mixed-effects model with PDM, food treatment, exposure time and their interactions as fixed effects and replicate as a random effect, followed by pairwise comparisons among PDMs and among food treatments.

Survival differed significantly among PDMs (*F*_2,936_ = 19.49, *p* < 0.001), among food treatments (*F*_3,936_ = 257.69, *p* < 0.001) and over time (*F*_1,936_ = 2660.40, *p* < 0.001), with significant predator × treatment (*F*_6,936_ = 3.36, *p* = 0.003) and predator × treatment × day interactions (*F*_6,936_ = 2.79, *p* = 0.011; Figure 3A–D). These interactions indicate that the trajectory of survival decline depended on the food treatment experienced by each PDM, rather than PDMs differing by a constant offset.

Food treatment was the dominant determinant of survival in all three PDMs (Figure 3E–G). Complete deprivation of both prey and pollen produced the most rapid decline, with no mites surviving beyond 8–10 days. Pollen alone extended survival to approximately 16–18 days but supported no PDMs to the end of the observation period. Both prey-containing treatments sustained survival substantially longer, confirming prey availability rather than pollen as the primary driver of persistence. The significant predator × treatment interaction reflected PDM differences in the additional benefit conferred by pollen when prey was available. In *N. californicus* and *N. barkeri*, survival under thrips-only and pollen-plus-thrips conditions was closely similar throughout, with all mites dying by approximately day 22 and day 26 respectively, indicating that pollen provided little further benefit once prey was present. In *A. largoensis*, by contrast, the two prey-containing treatments diverged from approximately day 8 onward and survival at day 30 was markedly higher when pollen was supplied alongside prey (approximately 22%) than when prey was provided alone (approximately 2%). *A. largoensis* was therefore the only PDM to derive additional benefit from pollen supplementation in the presence of prey and the only PDM with survivors remaining at the end of the 30-day period. Averaged across treatments, *A. largoensis* achieved the highest overall survival, followed by *N. barkeri* and then *N. californicus*. Pairwise comparisons confirmed that *A. largoensis* survived significantly better than both *N. californicus* (Δ = 18.3%, *p* < 0.001) and *N. barkeri* (Δ = 10.7%, *p* < 0.001) and that *N. barkeri* survived significantly better than *N. californicus* (Δ = 7.6%, *p* < 0.001).

### 3.4. Interspecific Interactions Among PDMs Under Prey-Present and Prey-Absent Conditions

**Figure 4:**
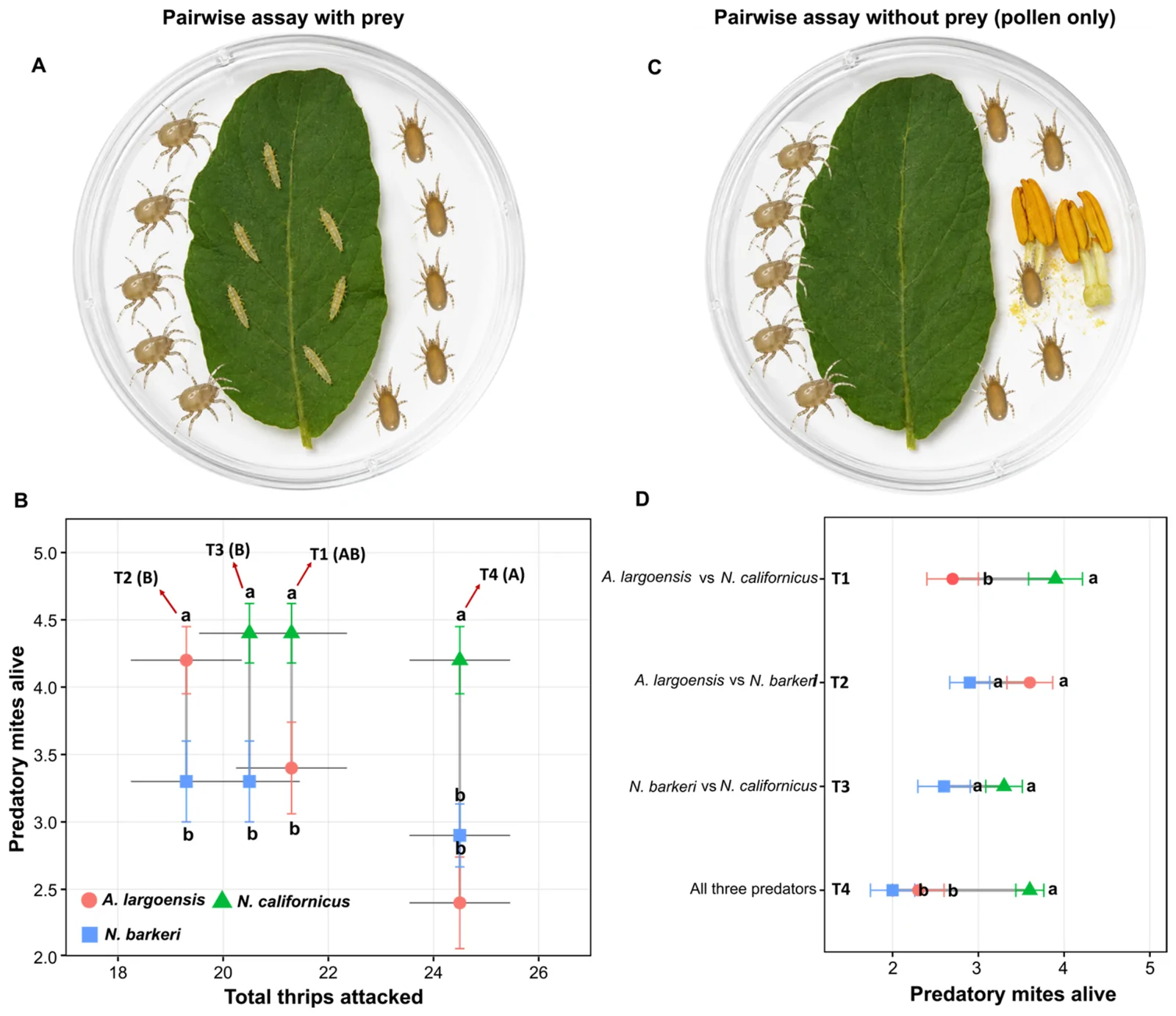
Survival and prey consumption of PDMs in mixed-PDM arenas under prey-present and prey-absent conditions. (**A**) Arena with *T. tabaci* prey only. (**B**) Total thrips attacked and number of PDMs surviving under each combination when only prey was available: T1, *A. largoensis* with *N. californicus*; T2, *A. largoensis* with *N. barkeri*; T3, *N. barkeri* with *N. californicus*; T4, all three PDMs combined. Uppercase letters indicate significant differences in prey attacked among combinations; lowercase letters indicate significant differences in survival among PDMs within each combination. (**C**) Arena with pollen only. (**D**) Number of PDMs surviving under each combination when only pollen was available. *n* = 10 replicate arenas per combination and condition. Points and error bars represent mean ± SE. Data were analyzed using binomial and quasi-binomial GLMs as appropriate.

When thrips prey was present, total prey attacked differed significantly among the four combinations (T1, *A. largoensis* with *N. californicus*; T2, *A. largoensis* with *N. barkeri*; T3, *N. barkeri* with *N. californicus*; T4, all three PDMs) (GLM: *F*_3,36_ = 4.91, *p* = 0.006; Figure 4B). When thrips prey was present, total prey attacked differed significantly among the four combinations (GLM: *F*_3,36_ = 4.91, *p* = 0.006; Figure 4B). The number attacked was the highest in T4, intermediate in T1, and the lowest and statistically indistinguishable between T2 and T3. Because T4 contained 15 mites whereas each pairwise combination contained 10, differences in total prey attacked between T4 and the pairwise combinations reflect predator number and are not interpreted further.

Predator survival under the same prey-present conditions revealed a hierarchy that did not mirror individual foraging performance. Although *A. largoensis* had shown the fastest foraging and the highest survival when tested alone, it survived significantly less well than *N. californicus* when the two were paired (T1: χ^2^_1_ = 6.00, *p* = 0.014). *A. largoensis* nevertheless survived significantly better than *N. barkeri* in direct pairing (T2: χ^2^_1_ = 4.40, *p* = 0.036) and *N. californicus* survived better than *N. barkeri* when those two were combined (T3: χ^2^_1_ = 7.06, *p* = 0.008). With all three PDMs together, survival again differed significantly (T4: χ^2^_2_ = 15.90, *p* < 0.001), following the order *N. californicus* > *A. largoensis* > *N. barkeri*. This ranking was consistent across T1, T3 and T4, identifying *N. californicus* as the PDM with the greatest survival advantage in mixed assemblages despite its comparatively weaker solo foraging speed and survival.

A broadly similar but attenuated pattern emerged under pollen-only conditions (Figure 4D). *N. californicus* again survived significantly better than *A. largoensis* (T1: χ^2^_1_ = 6.52, *p* = 0.011), indicating that this advantage was not contingent on prey availability. However, the difference between *A. largoensis* and *N. barkeri* was no longer significant in the absence of prey (T2: χ^2^_1_ = 2.16, *p* = 0.141), nor was that between *N. barkeri* and *N. californicus* (T3: χ^2^_1_ = 2.03, *p* = 0.154), indicating that the advantage of *A. largoensis* and *N. californicus* over *N. barkeri* was partly prey-dependent. Survival still differed significantly with all three PDMs combined (T4: χ^2^_2_ = 11.90, *p* = 0.003), with *N. californicus* again highest. Taken together, these results indicate that relative survival in mixed assemblages was not predicted by individual foraging or survival performance and that *N. californicus* consistently attained the highest survival across both pairwise and three-PDM assays regardless of prey availability.

## 4. Discussion

Phytoseiid PDMs used against thrips differ in the food resources they exploit and in how those resources sustain them, yet candidate agents are typically screened on predation rate against the target pest alone, with resource use and interactions among co-occurring predators assessed separately if at all [22,34,35]. Whether performance measured under one set of resource conditions predicts performance under another or when a second predator is present is therefore rarely tested within a single system [36,37]. The present study addresses this gap by comparing three phytoseiids of different origin, one indigenous PDM not developed for augmentative release and two commercially available species, across the traits that determine performance in the field: prey use, survival on prey and pollen and their combination, and interactions when sharing an arena with a competitor.

All three PDMs consumed early larval instars of *T. tabaci* at the highest rate, consistent with the general expectation that phytoseiids target the most vulnerable prey stages, which have reduced mobility, thinner cuticle and limited defensive capacity [38,39]. This has a direct practical implication, since releases timed to coincide with peak larval emergence rather than adult activity should yield the greatest suppression of population growth, although earlier release while adult thrips are still active may allow predators to establish before larval densities rise, so the optimal timing will depend on how quickly a released population builds. This distinction is reinforced by the spatial separation of the later stages. *T. tabaci* prepupae and pupae leave the foliage and complete development in the soil and litter, whereas the three PDMs evaluated here are foliage-dwelling, so these stages are largely inaccessible in the field even though they were present in our leaf arenas. The low attack rates recorded for prepupae and pupae therefore represent an upper bound on their contribution to suppression, and control by these predators depends almost entirely on the larval stages that remain on the plant [31,40]. Egg predation followed a different pattern, in which *N. californicus* consumed nearly all the eggs offered while *N. barkeri* consumed significantly fewer than were available and left most of them intact, with *A. largoensis* intermediate and statistically inseparable from either. Because phytoseiids cannot pierce leaf tissue, access to embedded eggs is restricted to the exposed chorion, so the differences recorded here reflect the capacity to exploit that limited access rather than egg predation in an unrestricted sense. Phytoseiids therefore differ in their capacity to intercept thrips recruitment at the egg stage, and this capacity is independent of performance against larvae, since *N. barkeri* attacked larval instars at rates comparable to *A. largoensis* [38].

The behavioral results reveal a more nuanced picture than foraging speed alone would suggest. *A. largoensis* located and attacked prey fastest in both confined arenas and on whole plants, yet retained each prey item longest and attacked fewer prey than either congener across the density gradient. Longer handling time alongside faster prey location is consistent with a strategy of thorough extraction per prey item rather than rapid serial attack [41,42,43], which would account for the lower number attacked despite the faster interception. Notably, this did not translate into weaker crop protection, since thrips-induced leaf damage was lowest in *A. largoensis* arenas despite the fewest larvae being attacked, with the three PDMs separating significantly from one another at every density from 20 prey upward. The explanation lies in when prey were attacked rather than how many were attacked. Because *A. largoensis* intercepted prey soonest after release, thrips in these arenas fed for a shorter period before being attacked, and the damage a thrips inflicts accumulates with the time it spends feeding rather than being fixed at the moment of encounter. A predator that reaches prey quickly therefore removes feeding time from every individual it encounters, whereas one that reaches prey slowly allows damage to accumulate even from larvae it eventually attacks. Non-consumptive disturbance may contribute further, since larvae that are approached and attacked without being killed are likely to interrupt feeding and relocate [44,45], though this was not measured directly. Speed of interception, rather than total number attacked, therefore appears the more relevant predictor of leaf-level damage.

Persistence during periods of low prey availability often determines whether early-season releases establish a resident predator population before pest pressure builds [46]. Prey availability, rather than pollen, was the dominant driver of survival in all three PDMs, consistent with the recognition that pollen functions as a supplementary rather than fully substitutable food for many phytoseiids [18,47]. Pollen alone extended survival by roughly a week relative to complete deprivation but supported no individual of any species to the end of the observation period, although potato pollen may be nutritionally suboptimal for phytoseiids and survival on a higher-quality pollen such as maize could well differ. The significant predator × treatment interaction, however, indicates that this generalization requires qualification at the species level. In both *Neoseiulus* PDMs, pollen conferred no detectable additional benefit once prey was present, but in *A. largoensis,* survival was substantially higher on pollen plus prey than on prey alone and this was the only PDM with survivors remaining at day 30. That the two resources acted additively in one species and not the others suggests the difference lies in how the resources are used rather than in whether pollen is accepted at all, since every species survived longer on pollen than on nothing. One possibility is that mixed diets relieve a nutritional constraint that prey alone does not satisfy, so that pollen supplies something complementary rather than merely additional calories; another is that pollen reduces the energetic cost of search when prey is present but dispersed, an effect that would be strongest in the species that searches most actively. Distinguishing these requires measurement of feeding rate and reproductive output on mixed diets rather than survival alone. Either way, the capacity to exploit both resources together is practically valuable, since it suggests that supplementary pollen provision in the field would extend persistence for *A. largoensis* specifically [48,49]. More broadly, the survival hierarchy, *A. largoensis* > *N. barkeri* > *N. californicus*, did not track foraging speed or egg predation. *N. californicus*, the most effective egg predator and second-fastest forager, showed the weakest starvation tolerance, while *N. barkeri*, the slowest forager, tolerated nutritional stress comparatively well. Foraging efficiency and starvation resistance therefore appear to be governed by distinct mechanisms, and field persistence cannot be reliably predicted from short-term foraging assays [50,51].

Performance measured in isolation did not predict relative survival when PDMs were combined [52,53]. Despite its superior foraging speed and solo survival, *A. largoensis* survived significantly less than *N. californicus* in direct pairing, both with prey present and with pollen alone. This asymmetry is specific: *A. largoensis* survived better than *N. barkeri* in direct pairing, so it is subordinate to *N. californicus* rather than competitively weak in general. Asymmetric interactions among phytoseiids are well documented and have been related to body size, developmental stage and aggressiveness [53,54,55], and the dissociation observed here suggests that the sensory and behavioral traits underlying prey location differ from those determining standing within a mixed guild [56]. The advantage of *A. largoensis* and *N. californicus* over *N. barkeri* was significant only when prey was present, indicating a partly prey-dependent asymmetry, whereas the advantage of *N. californicus* over *A. largoensis* persisted regardless of food availability. These assays measured net survival outcomes, and distinguishing intraguild predation from interference or differential stress tolerance will require direct behavioral observation [57,58,59].

Several constraints bound the inferences drawn here, beginning with the fact that performance was assessed over short intervals on detached leaves and potted plants, so field validation is a necessary next step, alongside assessment of fecundity and net reproductive rate on each food regime, which would establish whether the survival differences recorded here translate into differences in population growth, together with thermal tolerance and compatibility with the selective insecticides used alongside releases in integrated programs, which together determine whether a released population establishes [60,61,62]. A single potato cultivar was used throughout, so leaf surface features cannot account for the differences recorded, but whether these rankings hold on other hosts of this polyphagous pest remains untested. Two features of the design also limit specific comparisons: predator number was higher in the three-PDM treatment than in the pairwise combinations, so the greater total prey attacked there reflects predator density and cannot be attributed to species complementarity.

## 5. Conclusions

Selecting a phytoseiid agent on the basis of predatory capacity alone risks overlooking traits that determine performance once the predator is in the field, as the present comparison makes clear. *A. largoensis*, an indigenous PDM not developed for augmentative release, located and attacked *T. tabaci* faster and tolerated food limitation better than two commercially available species, yet attacked fewer prey across the density gradient and was consistently outcompeted by *N. californicus* in shared arenas, so that foraging efficiency and standing within a mixed predator assemblage emerge as dissociable traits, from which neither can be inferred from the other. Food resource use divided the three species along a further axis, since prey rather than pollen sustained survival in every PDM but only *A. largoensis* gained additional benefit from the two resources together, indicating that the capacity to exploit a mixed diet is species-specific rather than a general property of the family. Obtained under laboratory and glasshouse conditions, these findings identify *A. largoensis* as a candidate worth further evaluation and caution against selecting biological control agents on the basis of any single assay.

## Data Availability

The original contributions presented in this study are included in the article/Supplementary Material. Further inquiries can be directed to the corresponding authors.

## Supplementary Materials

The following supporting information can be downloaded at: https://www.mdpi.com/article/10.3390/insects17101074/s1, Figure S1. Experimental design for the prey-density assay used to assess density-dependent predation by three phytoseiid PDMs, Figure S2. Mean searching time required by PDMs to locate *T. tabaci* prey under confined Petri-dish conditions.

## Abbreviations

The following abbreviations are used in this manuscript:

PDM	predatory mite
GLM	generalized linear model
ANOVA	analysis of variance
HSD	honestly significant difference
