# Supplementary material for: Comparative Analysis of Thrips Prey and Pollen Usage by Three Predatory Mites, Amblyseius largoensis, Neoseiulus californicus and Neoseiulus barkeri
Source: Insects. 2026 Oct 17;17(10):1074. doi: 10.3390/insects17101074 (PMC13705122; doi:10.3390/insects17101074)
Supplement: Supplementary file 1 [file insects-17-01074-s001.zip › insects-4597373-supplementary.pdf]

Supporting Information's

Comparative analysis of thrips prey and pollen usage by three predatory mites, *Amblyseius largoensis*, *Neoseiulus californicus* and *Neoseiulus barkeri*

The following supporting information is available:

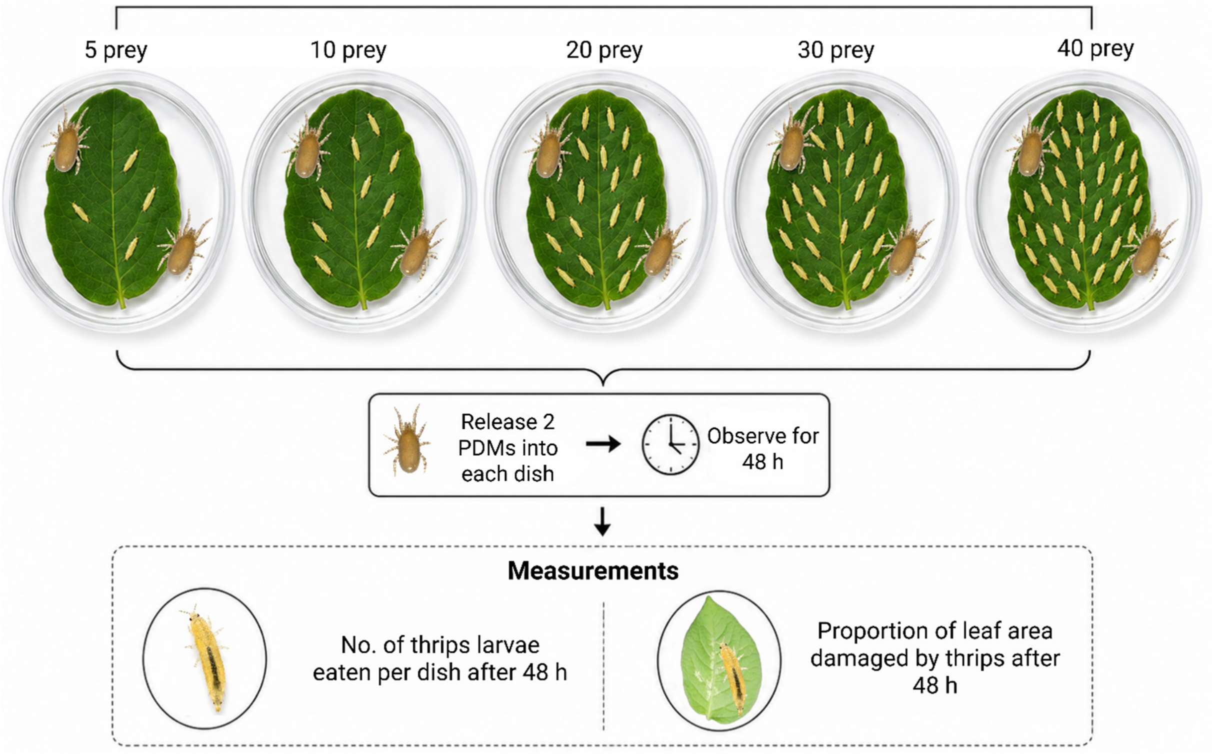

Figure S1. Experimental design for the prey-density assay used to assess density-dependent predation by three phytoseiid PDMs. Two PDMs of a given species were released onto a potato leaflet disc infested with *T. tabaci* larvae at one of five densities (5, 10, 20, 30 or 40 individuals per disc) and allowed to forage for 48 h, after which the number of prey attacked and the proportion of leaf area damaged were recorded for each replicate (n = 10 replicates per species at each density).

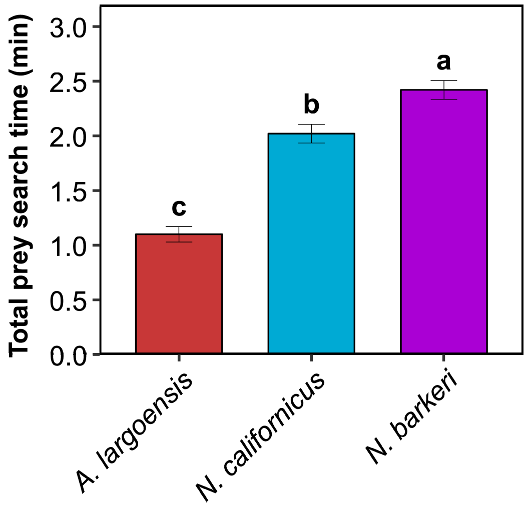

Figure S2. Mean searching time required by PDMs to locate *T. tabaci* prey under confined petri-dish conditions. Bars represent mean ± SE; different lowercase letters indicate statistically significant differences among predator species (one-way ANOVAs followed by Tukey's HSD post hoc test, as appropriate ($p < 0.05$).
